# Real-world safety of Tepotinib: Insights from the Food and Drug Administration Adverse Event Reporting System

**DOI:** 10.1371/journal.pone.0339005

**Published:** 2025-12-18

**Authors:** Su Wei, Lixi Zhang, Cuiping Liu, Xu Qi

**Affiliations:** Department of Respiratory and Critical Care Medicine, The First Affiliated Hospital of Nanjing Medical University, Nanjing, China; National University of Rosario, ARGENTINA

## Abstract

**Objects:**

Tepotinib is widely utilized for treating non-small cell lung cancer (NSCLC) patients with MET exon 14 (METex14) skipping mutations. The purpose of this study is to evaluate the safety characteristics of Tepotinib in the actual environment by analyzing data from the Food and Drug Administration Adverse Event Reporting System (FAERS) database.

**Methods:**

This study analyzed adverse event (AE) reports related to Tepotinib from FAERS database spanning Q1 2021 to Q4 2024. Four disproportionality analysis methods were employed: Reporting Odds Ratio (ROR), Proportional Reporting Ratio (PRR), Multi-Item Gamma Poisson Shrinker (MGPS), and Bayesian Confidence Propagation Neural Network (BCPNN). A descriptive analysis and a subgroup analysis of the time to onset (TTO) of AEs was conducted, and the Weibull distribution was used to predict temporal variations in AEs.

**Result:**

A total of 521 AE reports involving 1,385 AEs were included in this study. Of these, 69.50% were from the elderly population. The analysis confirmed several known AEs such as death, peripheral edema, diarrhea, and blood creatinine increased while also identifying previously unreported signals, including deafness and taste disorder. For females, special attention ought to be directed toward alopecia, deafness and abdominal pain upper. For males, the onset of interstitial lung disease, pruritus and constipation requires observation. The median time to onset (TTO) of AEs was 39 days, with 43.88% of AEs occurring in the first month of treatment initiation. Additionally, the individuals aged 75 years and over the experience AEs relatively early.

**Conclusion:**

This study conducted a comprehensive evaluation of the real-world safety of Tepotinib. The study validates clinical assertions about Tepotinib’s safety profile and identifies its novel signals, and also emphasized early monitoring of treatment. Our findings will better guide the clinical practice of Tepotinib and provide a basis for larger prospective studies in the future.

## Introduction

Lung cancer is one of the most aggressive and prevalent diseases, with an estimated 2.5 million new cases (12.4% of all cancers) and 1.8 million deaths (18.7% of all cancer deaths) in 2022 [1]. It is broadly categorized into small-cell and non-small-cell lung cancer (NSCLC), with the latter accounting for over 85% of cases. The most common histological subtypes of NSCLC are adenocarcinoma (40%) and squamous cell carcinoma (25%) [2]. In recent years, with the advent of the era of precision oncology, the field of pharmacological therapy for NSCLC has advanced quickly, and molecularly targeted therapy has exhibited remarkable efficacy. Patients with certain carcinogenic drivers, such as EGFR, ALK, HER2, ROS1, and more recently MET mutations, benefit greatly from targeted therapy [3]. The MET proto-oncogene encodes the tyrosine kinase receptor of hepatocyte growth factor (HGF). When the receptor binds to its ligand, MET can activate downstream signaling via the PI3K/ AKT/ mTOR and RAS/ RAF/ MEK/ ERK pathways, thereby driving cell proliferation and migration. The main mechanisms of the oncogenic activation include protein overexpression, gene mutation, amplification and rearrangement [4,5]. Among them, METex14 skipping mutations and MET amplification are the two most significant manifestations of MET pathway disorders. At present, METex14 skipping is the sole therapeutic target for NSCLC patients with MET pathway disorders, and its related targeted drugs such as carmatinib, tepatinib, and sevotinib have completely changed the treatment mode and prognosis of patients [6].

Tepotinib is an effective, highly MET-selective, and highly oral bioavailability small molecule tyrosine kinase inhibitor (TKI), which has been approved by the FDA on February 3, 2021 [7]. In the context of METex14 skipping mutation, preclinical evidence indicates that Tepotinib potently inhibits MET pathway-related phosphorylation, suppressing tumor cell proliferation, survival, and metastasis [8]. VISION (NCT02864992) is a global, single-arm, multi-cohort, open-label phase II trial designed to evaluate the anti-tumor activity and tolerance of Tepotinib in advanced NSCLC patients with METex14 skipping. According to the latest results, the overall response rate (ORR) was 51.4%, and the median progression-free survival (PFS) was 11.2 months. It is worth noting that Tepotinib exhibited a better response in treatment naive patients, with an ORR of 57.3% and a median PFS of 12.6 months. Among the patients in the trial, 34.8% had grade 3 or above adverse reactions, and fortunately, only 14.7% of the patients discontinued due to adverse reactions [9]. In addition, another phase II study (KCSG AL19−17) evaluating the efficacy and safety of Tepotinib in 35 patients with advanced solid tumors carrying MET exon 14 skipping mutations or amplification also showed good efficacy and safety [10]. However, considering the limitations such the scale of the subjects and strict screening criteria, the safety of Tepotinib in the real-world environment still needs further study.

The FDA Adverse Event Reporting System (FAERS) systematically collects data on adverse drug reactions and medication errors reported spontaneously by medical staff and consumers, playing a crucial role in post-marketing safety monitoring of drugs [11].

These real-world data can help researchers identify potential safety signals and effectively complement the deficiencies found in pre-marketing studies. The purpose of this study is to comprehensively evaluate the real-world safety of Tepotinib by conducting an extensive analysis of the FAERS database, and to provide preliminary safety insights for health care professionals.

## Materials and methods

### Data sources

The FAERS database covers all marketed pharmaceuticals and therapeutic biologics that are subject to the FDA’ s post-marketing safety surveillance program. In addition to reports directly from patients and healthcare professionals, it includes AE reports that the FDA has obtained from manufacturers as mandated by law. The FAERS database comprises seven document types: Demographic (DEMO), adverse event coding (REAC), Drug Information (DRUG), Patient outcomes (OUTC), Reporting Source (RPSR), Start and End Dates of Reported Drugs (THER), and Indications for use/diagnosis (INDI). Although serving as a spontaneous reporting system, which inherently contains missing or inaccurate data, FAERS is one of the world’s biggest databases for tracking adverse medication events. No informed consent or ethical approval was necessary for this study because FAERS data are publicly available and patient information in adverse reaction reports is anonymized.

### Data extraction

**Fig 1:**
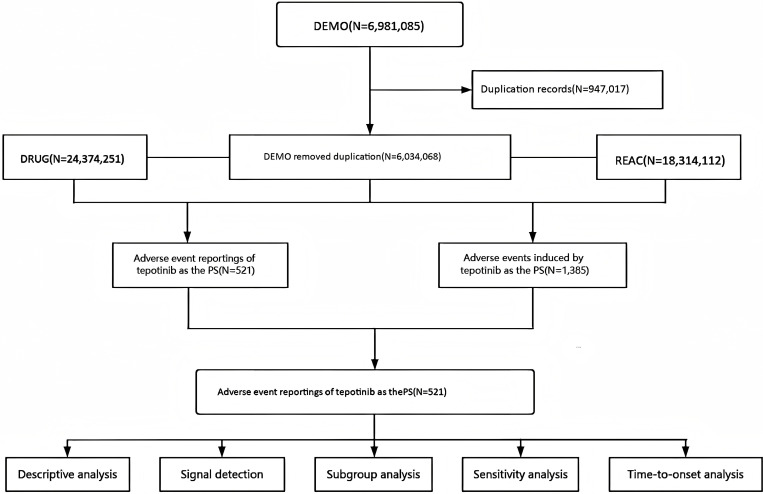
Flowchart depicting the process of analyzing AEs related to Tepotinib using the FDA Adverse Event Reporting System. Abbreviations: DEMO, demographics; DRUG, drug information; REAC, adverse events; PS, primary suspected.

The relationship between drugs and AEs can be classified as primary suspect (PS), secondary suspect (SS), concomitant (C), and interaction (I). To more comprehensively describe the relationship between Tepotinib and AEs, both the generic name “Tepotinib” and the trade name “Tepmetko” were used as PS in the analysis. The study period was chosen from Q1 2021, the time when Tepotinib was first approved, to Q4 2024, as this represents the latest available data from the FAERS database at the time of conducting this study. This time frame ensures that the most recent and relevant data was included in the analysis. Preferred Terms (PTs) from the standardized Medical Dictionary for Regulatory Activities 27.1 (MedDRA), which covers 27 system organ classes (SOCs), were used to code the Tepotinib adverse occurrences. To guarantee the quality of the data utilized for analysis, we identified and corrected errors, inconsistencies, and missing values after the raw data was collected. And for the sake of precise identification of potential safety signals, we concentrated on SOC and PT levels for our pharmacovigilance investigation of Tepotinib. The detailed data mining process was shown in Fig 1. Relevant clinical characteristics, such as gender, age, reporting region, reporter identity, and reporting duration, were carefully gathered and described in addition to the AEs data.

### Statistical analysis

#### Disproportionality analysis.

To explore the relationship between Tepotinib and AEs, this study used four descriptive statistical techniques to conduct a comprehensive analysis of the data, including Reporting Odds Ratio (ROR) [12], Proportional Reporting Ratio (PRR) [13], Bayesian Confidence Propagation Neural Network (BCPNN) [14], and Multi-Item Gamma Poisson Shrinkage (MGPS) [15]. The ROR and PRR approaches identify abnormally high adverse event reporting rates and highlight signals of AEs associated with Tepotinib. As a advanced algorithm, BCPNN is more adept at spotting potential connections between drug-adverse events. When measuring adverse event signals, the MGPS approach is more comprehensive since it considers both background hazards and the quantity of reports. All algorithms are based on a 2 × 2 matrix table (S1 Table), determined by standard formulas to assess potential connections between Tepotinib and AEs (S2 Table). Positive signals were defined as AEs that met the criteria of at least one disproportionality analysis method.

#### Time to Onset (TTO) and Weibull distribution analysis of AEs.

Time to Onset (TTO) for Tepotinib-related adverse events (AEs) was defined as the time interval between the AE onset date in the DEMO file and the drug start date in the THER file. For analytical convenience, all TTOs were consistently converted into days. We conducted a gender and age subgroup analysis of TTO. The Log-Rank test was used to analyze the differences among subgroups. Additionally, the Weibull distribution was applied to assess changes in the occurrence of AEs over time. All data processing and statistical analyses were performed using R (Version 4.4.1).

## Results

### Descriptive analysis

Throughout the surveillance period spanning from the first quarter of 2021 to the forth quarter of 2024, the FAERS database yielded a total of 6,981,085 reports. After strict selection and analysis, 521 AEs reports pertained to Tepotinib were analyzed. Among the Tepotinib-related AE reports, the impact was greater for female patients (n = 272, 52.2%) than for male patients (n = 232, 44.5%). In terms of age distribution, the largest proportion of reports originated from the elderly population (aged 65 and above), accounted for 69.5%. Due to the lack of weight data in most cases, we were unable to accurately analyze the basic characteristics of the drug-using crowd. Concerning AE outcomes, death reports (n = 142, 27.3%) were the most frequently documented, followed by other serious outcomes (n = 133, 21.7%) and hospitalization (n = 86, 16.5%). And a small number of other serious AEs, including life-threatening (n = 4, 0.8%) and disability conditions (n = 3, 0.6%), were reported. Since the FDA approved Tepotinib in 2021, the year with the fewest cases was 2021 (n = 68, 13.1%), while the year with the most cases was 2023(n = 208, 39.9%). Geographically, most adverse reaction reports came from United States (n = 256, 49.1%) and Japan (n = 118, 22.6%). Predominantly, reports were submitted by physician (n = 218, 41.8%) and consumers (n = 213, 40.9%). More detailed information on AE reports is provided in Table 1.

**Table 1:** Clinical characteristics of Tepotinib adverse event reports from the FAERS database (Q1 2021 - Q4 2024).

Characteristics	Case numbers	Case proportion (%)
Number of events	521	
**Gender**		
Male	232	44.5
Female	272	52.2
Miss	17	3.3
**Age**		
Median (IQR)	76.0 (70.0-81.0)	
<18	5	1
18-65	49	9.4
65-85	315	60.5
>85	47	9
Miss	105	20.2
**Top 5 Reported Countries**		
United States	256	49.1
Japan	118	22.6
United kingdom	18	3.5
Germany	15	2.9
Belgium	15	2.9
**Reporter**		
Physician	218	41.8
Consumer	213	40.9
Health professional	42	8.1
Pharmacist	30	5.8
Missing	18	3.5
**Outcomes**		
Death	142	27.3
Other serious outcomes	113	21.7
Hospitalization	86	16.5
life-threatening	4	0.8
Disability conditions	3	0.6
Missing	173	33.2
**Reporting year**		
2021	68	13.1
2022	104	20
2023	208	39.9
2024	141	27.1

Abbreviation: interquartile range, IQR.

### Distribution of AEs at the SOC level

**Fig 2:**
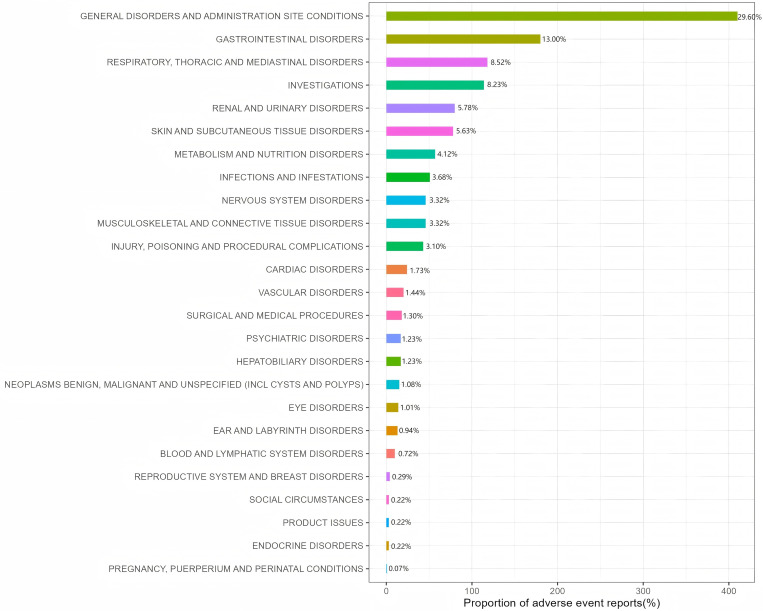
Adverse event distribution across system organ classes for Tepotinib.

A total of 25 SOCs were recorded in AE reports related to Tepotinib, with specific signal strengths shown in Table 2. The positive reported SOCs included general disorders and administration site conditions, gastrointestinal disorders, investigations, respiratory, thoracic and mediastinal disorders, investigations, renal and urinary disorders, metabolism and nutrition disorders and ear and labyrinth disorders. Among them, renal and urinary disorders was the sole SOC that met all four algorithms simultaneously. In addition, ear and labyrinth disorders were not recorded on the drug label. Fig 2 visually presents the distribution of AEs at the SOC level.

**Table 2:** Signal strength of Tepotinib AEs across System Organ Classes (SOC) in the FAERS database.

System Organ Class (SOC)	Case numbers	ROR (95%CI)	PRR (^2^)	EBGM(EBGM05)	IC(IC025)
General Disorders And Administration Site Conditions*	410	1.96 (1.74-2.19)	1.67 (134.78)	1.67 (1.52)	0.74 (0.58)
Gastrointestinal Disorders*	180	1.78 (1.52-2.08)	1.68 (53.20)	1.68 (1.47)	0.74 (0.52)
Respiratory, Thoracic And Mediastinal Disorders*	118	1.99 (1.65-2.40)	1.91 (53.10)	1.90 (1.63)	0.93 (0.65)
Investigations*	114	1.46 (1.21-1.77)	1.42 (15.33)	1.42 (1.21)	0.51 (0.23)
Renal And Urinary Disorders*	80	3.48 (2.78-4.37)	3.34 (133.46)	3.34 (2.76)	1.74 (1.41)
Skin And Subcutaneous Tissue Disorders	78	1.09 (0.87-1.37)	1.08 (0.53)	1.08 (0.90)	0.12 (−0.22)
Metabolism And Nutrition Disorders*	57	2.23 (1.71-2.91)	2.18 (37.28)	2.18 (1.75)	1.13 (0.74)
Infections And Infestations	51	0.63 (0.47-0.83)	0.64 (10.83)	0.64 (0.51)	−0.64 (−1.05)
Nervous System Disorders	46	0.45 (0.34-0.60)	0.47 (29.95)	0.47 (0.37)	−1.10 (−1.52)
Musculoskeletal And Connective Tissue Disorders	46	0.64 (0.48-0.86)	0.65 (9.05)	0.65 (0.51)	−0.62 (−1.05)
Injury, Poisoning And Procedural Complications	43	0.21 (0.16-0.29)	0.24 (120.05)	0.24 (0.19)	−2.07 (−2.51)
Cardiac Disorders	24	0.92 (0.61-1.37)	0.92 (0.17)	0.92 (0.66)	−0.12 (−0.70)
Vascular Disorders	20	0.79 (0.51-1.23)	0.8 (1.05)	0.80 (0.55)	−0.33 (−0.96)
Surgical And Medical Procedures	18	0.85 (0.53-1.35)	0.85 (0.49)	0.85 (0.58)	−0.24 (−0.90)
Psychiatric Disorders	17	0.22 (0.14-0.36)	0.23 (45.81)	0.23 (0.16)	−2.11 (−2.80)
Hepatobiliary Disorders	17	1.49 (0.93-2.41)	1.49 (2.75)	1.49 (1.00)	0.57 (−0.11)
Neoplasms Benign, Malignant And Unspecified (Incl Cysts And Polyps)	15	0.25 (0.15-0.42)	0.26 (32.32)	0.26 (0.17)	−1.93 (−2.65)
Eye Disorders	14	0.51 (0.30-0.86)	0.51 (6.61)	0.51 (0.33)	−0.96 (−1.71)
Ear And Labyrinth Disorders*	13	2.37 (1.37-4.10)	2.36 (10.24)	2.36 (1.49)	1.24 (0.46)
Blood And Lymphatic System Disorders	10	0.41 (0.22-0.77)	0.42 (8.33)	0.42 (0.25)	−1.26 (−2.14)
Reproductive System And Breast Disorders	4	0.51 (0.19-1.35)	0.51 (1.93)	0.51 (0.22)	−0.98 (−2.27)
Endocrine Disorders	3	0.80 (0.26-2.49)	0.80 (0.15)	0.80 (0.31)	−0.32 (−1.76)
Social Circumstances	3	0.46 (0.15-1.43)	0.46 (1.89)	0.46 (0.18)	−1.12 (−2.56)
Product Issues	3	0.11 (0.03-0.34)	0.11 (22.05)	0.11 (0.04)	−3.19 (−4.63)
Pregnancy, Puerperium And Perinatal Conditions	1	0.23 (0.03-1.61)	0.23 (2.64)	0.23 (0.04)	−2.14 (−4.18)

Abbreviation: Asterisks (*) indicate statistically significant signals in algorithm; ROR, reporting odds ratio; PRR, proportional reporting ratio; EBGM, empirical Bayesian geometric mean; EBGM05, the lower limit of the 95% CI of EBGM; IC, information component; IC025, the lower limit of the 95% CI of the IC; CI, confidence interval; AEs, adverse events.

### Distribution of AEs at the PT level

A thorough signal detection investigation revealed 71 positive PTs, with Table 3 depicting the top 50 AEs ranked by frequency among the positive signals. Common AEs including death, oedema peripheral, diarrhea, nausea and blood creatinine increased were consistent with established drug AE warnings. Notably, we identified some unexpected signals that were not mention in the safety profile: deafness and taste disorder. Additionally, all AEs that met the positive signal criteria are listed in S3 Table.

**Table 3:** Top 50 frequency of adverse events at the PT level for Tepotinib.

PT	Case numbers	ROR (95%CI)	PRR (^2^)	EBGM(EBGM05)	IC(IC025)
Death	107	6.06 (4.98-7.38)	5.67 (417.05)	5.67 (4.81)	2.50 (2.21)
Oedema Peripheral	61	38.47 (29.75-49.75)	36.82 (2122.43)	36.72 (29.62)	5.20 (4.82)
Diarrhoea	51	3.59 (2.71-4.74)	3.49 (91.60)	3.49 (2.76)	1.80 (1.40)
Fatigue	45	2.55 (1.90-3.43)	2.50 (41.04)	2.50(1.95)	1.32 (0.89)
Oedema	42	43.24 (31.79-58.82)	41.96 (1675.34)	41.83 (32.34)	5.39 (4.94)
Renal Impairment	42	21.67 (15.93-29.46)	21.04 (801.50)	21.01 (16.24)	4.39 (3.95)
Disease Progression	40	13.2 (9.64-18.08)	12.85 (437.52)	12.83 (9.86)	3.68 (3.22)
Nausea	38	2.52 (1.83-3.48)	2.48 (34.01)	2.48 (1.90)	1.31 (0.84)
Peripheral Swelling	25	6.05 (4.07-8.99)	5.96 (103.49)	5.96 (4.28)	2.58 (2.00)
Blood Creatinine Increased	23	18.65 (12.35-28.17)	18.36 (377.26)	18.33 (12.98)	4.20 (3.60)
Decreased Appetite	23	4.50 (2.98-6.80)	4.45 (61.62)	4.44 (3.15)	2.15 (1.56)
Vomiting	15	1.69 (1.01-2.81)	1.68 (4.16)	1.68 (1.10)	0.75 (0.02)
Pleural Effusion	13	12.07 (6.99-20.85)	11.97 (130.64)	11.96 (7.57)	3.58 (2.80)
Constipation	13	2.72 (1.58-4.70)	2.71 (14.04)	2.71 (1.71)	1.44 (0.66)
Interstitial Lung Disease	12	11.52 (6.52-20.33)	11.43 (114.14)	11.42 (7.09)	3.51 (2.71)
Weight Increased	11	2.38 (1.31-4.30)	2.36 (8.69)	2.36 (1.44)	1.24 (0.40)
Swelling	10	4.42 (2.37-8.23)	4.39 (26.24)	4.39 (2.61)	2.13 (1.26)
Generalised Oedema	9	39.66 (20.57-76.47)	39.41 (335.98)	39.30 (22.69)	5.30 (4.38)
Fluid Retention	9	9.70 (5.03-18.68)	9.64 (69.71)	9.64 (5.57)	3.27 (2.35)
Hypoalbuminaemia	8	50.34 (25.09-100.99)	50.05 (383.18)	49.87 (27.85)	5.64 (4.67)
Pulmonary Toxicity	8	40.3 (20.09-80.84)	40.08 (303.93)	39.96 (22.32)	5.32 (4.35)
Pulmonary Oedema	7	8.67 (4.12-18.22)	8.63 (47.20)	8.62 (4.63)	3.11 (2.08)
Alanine Aminotransferase Increased	7	7.01 (3.33-14.73)	6.98 (35.84)	6.97 (3.74)	2.80 (1.78)
Lung Disorder	7	6.57 (3.13-13.82)	6.55 (32.90)	6.54 (3.51)	2.71 (1.69)
General Physical Health Deterioration	7	2.52 (1.20-5.29)	2.51 (6.37)	2.51 (1.35)	1.33 (0.30)
Dry Skin	7	2.36 (1.13-4.97)	2.36 (5.49)	2.36 (1.27)	1.24 (0.21)
Pneumonitis	6	9.14 (4.10-20.39)	9.11 (43.29)	9.10 (4.65)	3.19 (2.09)
Aspartate Aminotransferase Increased	6	7.12 (3.19-15.88)	7.09 (31.40)	7.09 (3.62)	2.83 (1.73)
Renal Failure	6	2.48 (1.11-5.52)	2.47 (5.26)	2.47 (1.26)	1.30 (0.21)
Lymphoedema	5	29.81 (12.38-71.81)	29.71 (138.42)	29.64 (14.21)	4.89 (3.71)
Metastases To Central Nervous System	5	17.12 (7.11-41.22)	17.06 (75.53)	17.04 (8.17)	4.09 (2.91)
Pneumothorax	5	14.62 (6.07-35.19)	14.57 (63.14)	14.55 (6.98)	3.86 (2.68)
Blood Sodium Decreased	5	13.85 (5.75-33.36)	13.81 (59.36)	13.80 (6.61)	3.79 (2.60)
Deafness	5	9.02 (3.75-21.70)	8.99 (35.48)	8.98 (4.31)	3.17 (1.99)
Taste Disorder	5	5.68 (2.36-13.66)	5.66 (19.19)	5.66 (2.71)	2.50 (1.32)
Renal Disorder	5	5.10 (2.12-12.27)	5.08 (16.40)	5.08 (2.44)	2.34 (1.16)
Respiratory Failure	5	4.11 (1.71-9.89)	4.10 (11.71)	4.10 (1.96)	2.03 (0.85)
Hepatic Enzyme Increased	5	3.05 (1.27-7.35)	3.05 (6.87)	3.04 (1.46)	1.61 (0.43)
Blood Albumin Decreased	4	34.43 (12.89-92)	34.34 (129.15)	34.25 (15.05)	5.10 (3.80)
Nephropathy Toxic	4	15.64 (5.86-41.75)	15.59 (54.58)	15.58 (6.85)	3.96 (2.67)
Hospice Care	4	12.28 (4.60-32.79)	12.25 (41.31)	12.24 (5.38)	3.61 (2.32)
Pericardial Effusion	4	8.78 (3.29-23.44)	8.76 (27.49)	8.76 (3.85)	3.13 (1.84)
Hepatotoxicity	4	7.42 (2.78-19.79)	7.40 (22.12)	7.39 (3.25)	2.89 (1.59)
Hepatic Function Abnormal	4	4.84 (1.81-12.91)	4.83 (12.14)	4.82 (2.12)	2.27 (0.98)
Swelling Face	4	3.53 (1.32-9.43)	3.53 (7.24)	3.52 (1.55)	1.82 (0.52)
Cardiac Failure	4	2.49 (0.93-6.63)	2.48 (3.54)	2.48 (1.09)	1.31 (0.02)
Malignant Pleural Effusion	3	100.62 (32.27-313.72)	100.41 (293.03)	99.66 (38.49)	6.64 (5.19)
Infectious Pleural Effusion	3	74.29 (23.85-231.36)	74.13 (215.24)	73.72 (28.50)	6.20 (4.75)
Organising Pneumonia	3	24.32 (7.83-75.59)	24.27 (66.82)	24.23 (9.38)	4.60 (3.15)
Musculoskeletal Chest Pain	3	9.52 (3.07-29.58)	9.51 (22.82)	9.50 (3.68)	3.25 (1.80)

Abbreviation: ROR, reporting odds ratio; PRR, proportional reporting ratio; EBGM, empirical Bayesian geometric mean; EBGM05, the lower limit of the 95% CI of EBGM; IC, information component; IC025, the lower limit of the 95% CI of the IC; CI, confidence interval; PT, preferred term.

### Subgroup analysis

Gender subgroup analysis revealed that among the 20 most frequently reported positive AEs, alopecia, deafness, and abdominal pain upper were exclusively observed in females, whereas interstitial lung disease, pruritus, and constipation were solely reported in males. Detailed information can be found in S4 Table and S5 Table. Our age subgroup analysis showed that among the 20 most commonly reported positive AEs, only alopecia, pruritus, and anemia were observed in individuals under 75 years old, while dry skin, upper abdominal pain, and taste disorder occurred only in individuals aged 75 years and older. Specific details are available in S6 Table and S7 Table.

### TTO and Weibull distribution analysis of Tepotinib-related AEs

**Fig 3:**
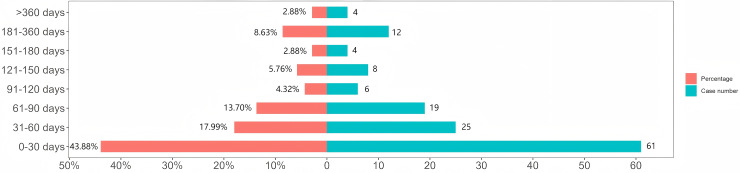
Time to onset of AEs associated with Tepotinib.

**Fig 4:**
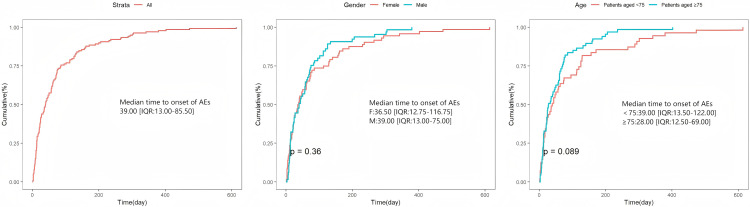
Time to onset of AEs associated with Tepotinib in subgroup analysis.

Comprehensive and precise details on the time of occurrence was provided for 139 AEs that were reported from the Faers database. As Fig 3 visualized, the onset of AEs was mainly concentrated in the first three months. In detail, the majority of AE instances took place in the first month (n = 61, 43.88%), second month (n = 25,17.99%) and third month (n = 19,13.67%) after Tepotinib initiation. The median onset time of these AEs was 39 days (interquartile range [IQR] 13.00–85.50 days). Based on the current sample scale, there was no statistical significance in the p-value of either gender or age subgroup. The median onset time of AEs in men and women was roughly similar. However, the median onset time of AEs in individuals aged 75 and over was 11 days shorter than that in individuals under 75 years old. The detailed results can be seen in Fig 4. Calculated shape parameter (β) of 0.83 with a 95% confidence interval (CI) range of 0.73 to 0.94 was found by evaluating the Weibull Shape Parameter analysis (Table 4). Notably, the Weibull Distribution result was a early-failure model, indicating the incidence of AEs was trending downward over time.

**Table 4:** Weibull distribution analysis of Tepotinib-associated adverse events.

Drug	Weibull distribution		
	Scale parameter: α(95%CI)	Shape parameter: β(95%CI)	Type
Tepotinib	68.52(54.03-83.00)	0.83(0.73-0.94)	Early failure

Abbreviation: CI, confidence interval; IQR, interquartile rang.

## Discussion

This study conducted a comprehensive evaluation of AEs associated with Tepotinib since its market launch in 2021. We confirmed AEs listed on the drug label, including death, oedema peripheral, diarrhoea, fatigue and blood creatinine increased. Furthermore, novel signals such as deafness and taste disorder were identified. These findings offer insights into the real-world safety of Tepotinib, thus guiding its safer and more informed use in clinical practice.

In our study, death and peripheral edema were identified as the two most frequently reported AEs, consistent with the safety profile in the FDA drug label. The predominance of death is likely due to the MET exon 14 skipping mutation, which has been shown to be an independent prognostic factor for reduced survival in cancer patients [16]. Peripheral edema is a well-known AE listed on the drug label. In a long-term follow-up report of VISION, the incidence of peripheral edema was 67.1% [9]. And the analysis of Asian patients from VISION showed that peripheral edema was still the most common AE [17]. These findings are consistent with the results of this study. Although the exact mechanism of oedema has not been confirmed, some researchers believe that a potential mechanism is that the HGF/MET signaling pathway is inhibited, hindering the role of HGF in protecting vascular endothelium from excessive permeability damage caused by vascular endothelial growth factor (VEGF) [18]. In addition, patients with oedema may experience negative feelings such as pain, swelling, heaviness, and paresthesia of limbs. At the same time, the overall health status will also be affected, which increases the complexity of cancer patient management [19]. Therefore, prevention, early identification and intervention of oedema are particularly important. We recommend that healthcare professionals regularly assess the patients’ weight and limb circumference and consider appropriate interventions such as exercise therapy, compression stockings, and diuretics.

Blood creatinine increased is another known common adverse reaction, which has been confirmed by several studies [17,20]. It is worth noting that some preclinical studies have indicated that Tepotinib-related blood creatinine increased is a pseudo-renal injury. The potential mechanism of this pseudo-renal injury may involve the competitive inhibition of creatinine transporters in renal tubules. Specifically, organic anion transporter 2 (OAT2) is responsible for the basolateral absorption of creatinine, while MATE1 and MATE2-K (apically expressed multidrug and toxin efflux transporters) mediate the secretion of creatinine into urine. Tepotinib may lead to an increase in serum creatinine levels by inhibiting the function of these creatinine tubule transporters [21,22]. However, a clinical case from Japan reported confirmed tubulointerstitial injury following Tepotinib treatment, indicating a risk of true renal injury [23]. Therefore, reatinine levels in patients treated with tepotinib need to be carefully monitored over a long period of time to prevent further complications or deterioration of the original disease. We suggest that utilizing cystatin C, a renal function marker that is not affected by tubular transporter inhibitors, to estimate glomerular filtration rate (GFR) can help distinguish between true renal injury and pseudo-renal injury [24].

Additionally, we identified several positive signals not mentioned: deafness and taste disorders. Deafness, as an unexpected AE, reached a positive threshold in all four imbalance analyses. Notably, drug-induced ototoxicity was also mentioned in the report of AEs of Capmatinib, another highly MET-selective inhibitor [25]. This suggests that ear and labyrinth disorders may be a potential class effect of MET inhibitors. Researchers believe that the pharmacological effects of MET inhibitors can lead to an unstable HGF/c-MET signaling pathway, which may cause deafness in patients by disrupting the development of stria vascularis. The stria vascularis is a non-sensory structure existing in the cochlear duct, which plays an important role in the balance of cochlear ions, the generation of cochlear potential and the formation of blood labyrinth barrier (BLB) [26]. The stria vascularis mainly consists of three types of cells: marginal cells, intermediate cells, and basal cells. It is worth noting that the intermediate cell layer is formed by the migration, differentiation and integration of melanocytes into the cochlea epithelium, and its loss has been shown to be associated with deafness and hair cell loss. More and more studies have shown that defects in the HGF/c-MET signaling pathway can lead to the inability of melanocytes to successfully integrate the intermediate cell layer, resulting in hearing impairment [27,28]. In addition, a study focused on the protective effect of HGF on aminoglycoside-induced ototoxicity, suggesting that it may help protect hair cells from death [29]. In summary, the unsteady HGF/c-MET signaling pathway may increase the risk of hearing loss by impairing the development of non-sensory cochlear structures and reducing ear protection. Previous studies have shown that hearing loss is related to cognitive dysfunction, social isolation behavior and depression, which may lead to a decline in patient compliance and further lead to poor quality of life [30–32]. In view of the potential negative effects, clinicians should monitor for ototoxicity and the psychological well-being of patients receiving tepotinib, with timely intervention as needed.

Taste disorders emerged as novel AEs in this study. As the symptons are generally not life-threatening, they are often underestimated during cancer treatment. However, there have been studies showing that patients with taste disorders not only have loss of appetite, weight loss and malnutrition, but also are more likely to have significant neuropsychological symptoms, such as anxiety, depression and sleep disorders [33]. This underscores the necessity for comprehensive clinical assessment beyond mere symptom reporting. Taste disorders are usually associated with chemotherapy and radiotherapy [34]. And the leading hypotheses include direct gustatory cytotoxicity [35,36], oral mucositis and zinc deficiency [37,38].In addition, a study of mice treated by vismodegib, a targeted drug for the treatment of basal-cell carcinoma, indicated that vismodegib can cause a significant reduction in the size of taste buds and the number of taste cells per taste bud, as well as the decrease also included Ki67 and Shh (Sonichedgehog) expressing cells in taste buds. At the same time, phospholipase Cβ2 and α-gustducin-expressing cells expressing cells responsible for sensing sweetness and bitterness, and T1R3, glucagon-like peptide-1 and glucagon expressing cells responsible for regulating sweetness sensitivity also experience reduction [39]. Nevertheless, future research is required to further reveal the potential relationship between tepotinib and taste disorders.

Gender-related subgroup analysis indicated that alopecia, deafness and abdominal pain upper require particular attention in females, while interstitial lung disease, pruritus and constipation may need to be noted in males. The occurrence of pruritus and anemia may be required to be monitored in patients under 75 years old, while dry skin and taste disorder may be advisable to be the focus of attention in those aged 75 years and over.

These findings provide new insights into the clinical application of Tepotinib in different genders and age groups, but further studies are needed to elucidate its underlying mechanisms.

Among the 139 AE reports obtained, the median onset time of Tepotinib-related AEs was 39 days (IQR = 13.00–85.50 days). Most AEs occurred within the first three months, with the highest incidence in the first month (n = 61, 43.9%). In this study, no significant effect of gender and age subgroups on the risk of AEs was found. It is worth noting that the median onset time of AEs in patients aged 75 years and over was 11 days shorter than that in patients under 75 years, suggesting that we still need to treat elderly patients with caution and incorporate more sample sizes in future studies to explore differences. In addition, the Weibull distribution model was used to simulate the occurrence of AE, and the results were consistent with the early failure model, indicating that the possibility of drug-induced AE decreased over time.

Even with the benefits of large-scale real-world research and the data mining methods utilized herein, there are still some existing constraints that should be carefully taken into account. First, the Faers database may naturally include incomplete or inaccurate data because it is a spontaneous reporting system that is submitted by groups such as consumers and physicians. Second, in view of the sample size of the subgroup study, our subgroup analysis is only based on the descriptive results of the current data. In the future, data of gender and age subgroups need to be further collected to further investigate these differences. Moreover, the Faers database was mainly derived from case reports. The disproportionality analysis carried out here did not determine risk or prove causation, but it did help show the strength of the AE signal. More research is required to elucidate the mechanisms of AEs and related risk factors in order to validate the possible adverse effects identified in this study. Notwithstanding the limitations above, our results act as an essential tool for medical practitioners, helping vigilantly monitor patients and ascertain the related AEs of Tepotinib.

## Conclusion

This study conducted a comprehensive evaluation of the real-world safety of Tepotinib based on the FAERS database. The study confirmed several known AEs, such as death, peripheral edema, and blood creatinine increased, requiring certain precautions to reduce the risk of medication. In addition, our analysis revealed some novel signals, including deafness and taste disorders. The cumulative incidence of AEs reached 43.88% within the first month of treatment, underscoring the necessity for intensive monitoring during this initial phase. This evidence provides initial real-world insights to guide safer clinical decision-making regarding Tepotinib. Further validation through large-scale prospective studies is warranted to substantiate these findings.

## Supporting information

S1 Table Two-by-two contingency table for disproportionality analyses. (DOCX)

S2 Table Four major algorithms used for signal detection. (DOCX)

S3 Table All adverse events meeting the positive signal threshold at the PT level. (DOCX)

S4 Table Top 20 most common positive adverse events of Tepotinib in males at the PT level. (DOCX)

S5 Table Top 20 most common positive adverse events of Tepotinib in females at the PT level. (DOCX)

S6 Table Top 20 most common positive adverse events of Tepotinib in patients under 75 years at the PT level. (DOCX)

S7 Table Top 20 most common positive adverse events of Tepotinib in patients aged 75 years and over at the PT level. (DOCX)
