# Supplementary material for: Real-world safety of Tepotinib: Insights from the Food and Drug Administration Adverse Event Reporting System
Source: PLoS One. 2025 Dec 18;20(12):e0339005. doi: 10.1371/journal.pone.0339005 (PMC12714243; doi:10.1371/journal.pone.0339005)
Supplement: S3 Table — (DOCX) [file pone.0339005.s003.docx]

Supporting information

**S3 Table. All adverse events meeting the positive signal threshold at the PT level.**

| PT | Case numbers | ROR(95%CI) | PRR(χ^2^) | EBGM(EBGM05) | IC(IC025) |
| --- | --- | --- | --- | --- | --- |
| Death | 107 | 6.06 ( 4.98 - 7.38 ) | 5.67 ( 417.05 ) | 5.67 ( 4.81 ) | 2.5 ( 2.21 ) |
| Oedema Peripheral | 61 | 38.47 ( 29.75 - 49.75 ) | 36.82 ( 2122.43 ) | 36.72 ( 29.62 ) | 5.2 ( 4.82 ) |
| Diarrhoea | 51 | 3.59 ( 2.71 - 4.74 ) | 3.49 ( 91.6 ) | 3.49 ( 2.76 ) | 1.8 ( 1.4 ) |
| Fatigue | 45 | 2.55 ( 1.9 - 3.43 ) | 2.5 ( 41.04 ) | 2.5 ( 1.95 ) | 1.32 ( 0.89 ) |
| Oedema | 42 | 43.24 ( 31.79 - 58.82 ) | 41.96 ( 1675.34 ) | 41.83 ( 32.34 ) | 5.39 ( 4.94 ) |
| Renal Impairment | 42 | 21.67 ( 15.93 - 29.46 ) | 21.04 ( 801.5 ) | 21.01 ( 16.24 ) | 4.39 ( 3.95 ) |
| Disease Progression | 40 | 13.2 ( 9.64 - 18.08 ) | 12.85 ( 437.52 ) | 12.83 ( 9.86 ) | 3.68 ( 3.22 ) |
| Nausea | 38 | 2.52 ( 1.83 - 3.48 ) | 2.48 ( 34.01 ) | 2.48 ( 1.9 ) | 1.31 ( 0.84 ) |
| Peripheral Swelling | 25 | 6.05 ( 4.07 - 8.99 ) | 5.96 ( 103.49 ) | 5.96 ( 4.28 ) | 2.58 ( 2 ) |
| Blood Creatinine Increased | 23 | 18.65 ( 12.35 - 28.17 ) | 18.36 ( 377.26 ) | 18.33 ( 12.98 ) | 4.2 ( 3.6 ) |
| Decreased Appetite | 23 | 4.5 ( 2.98 - 6.8 ) | 4.45 ( 61.62 ) | 4.44 ( 3.15 ) | 2.15 ( 1.56 ) |
| Vomiting | 15 | 1.69 ( 1.01 - 2.81 ) | 1.68 ( 4.16 ) | 1.68 ( 1.1 ) | 0.75 ( 0.02 ) |
| Pleural Effusion | 13 | 12.07 ( 6.99 - 20.85 ) | 11.97 ( 130.64 ) | 11.96 ( 7.57 ) | 3.58 ( 2.8 ) |
| Constipation | 13 | 2.72 ( 1.58 - 4.7 ) | 2.71 ( 14.04 ) | 2.71 ( 1.71 ) | 1.44 ( 0.66 ) |
| Interstitial Lung Disease | 12 | 11.52 ( 6.52 - 20.33 ) | 11.43 ( 114.14 ) | 11.42 ( 7.09 ) | 3.51 ( 2.71 ) |
| Weight Increased | 11 | 2.38 ( 1.31 - 4.3 ) | 2.36 ( 8.69 ) | 2.36 ( 1.44 ) | 1.24 ( 0.4 ) |
| Swelling | 10 | 4.42 ( 2.37 - 8.23 ) | 4.39 ( 26.24 ) | 4.39 ( 2.61 ) | 2.13 ( 1.26 ) |
| Generalised Oedema | 9 | 39.66 ( 20.57 - 76.47 ) | 39.41 ( 335.98 ) | 39.3 ( 22.69 ) | 5.3 ( 4.38 ) |
| Fluid Retention | 9 | 9.7 ( 5.03 - 18.68 ) | 9.64 ( 69.71 ) | 9.64 ( 5.57 ) | 3.27 ( 2.35 ) |
| Hypoalbuminaemia | 8 | 50.34 ( 25.09 - 100.99 ) | 50.05 ( 383.18 ) | 49.87 ( 27.85 ) | 5.64 ( 4.67 ) |
| Pulmonary Toxicity | 8 | 40.3 ( 20.09 - 80.84 ) | 40.08 ( 303.93 ) | 39.96 ( 22.32 ) | 5.32 ( 4.35 ) |
| Pulmonary Oedema | 7 | 8.67 ( 4.12 - 18.22 ) | 8.63 ( 47.2 ) | 8.62 ( 4.63 ) | 3.11 ( 2.08 ) |
| Alanine Aminotransferase Increased | 7 | 7.01 ( 3.33 - 14.73 ) | 6.98 ( 35.84 ) | 6.97 ( 3.74 ) | 2.8 ( 1.78 ) |
| Lung Disorder | 7 | 6.57 ( 3.13 - 13.82 ) | 6.55 ( 32.9 ) | 6.54 ( 3.51 ) | 2.71 ( 1.69 ) |
| General Physical Health Deterioration | 7 | 2.52 ( 1.2 - 5.29 ) | 2.51 ( 6.37 ) | 2.51 ( 1.35 ) | 1.33 ( 0.3 ) |
| Dry Skin | 7 | 2.36 ( 1.13 - 4.97 ) | 2.36 ( 5.49 ) | 2.36 ( 1.27 ) | 1.24 ( 0.21 ) |
| Pneumonitis | 6 | 9.14 ( 4.1 - 20.39 ) | 9.11 ( 43.29 ) | 9.1 ( 4.65 ) | 3.19 ( 2.09 ) |
| Aspartate Aminotransferase Increased | 6 | 7.12 ( 3.19 - 15.88 ) | 7.09 ( 31.4 ) | 7.09 ( 3.62 ) | 2.83 ( 1.73 ) |
| Renal Failure | 6 | 2.48 ( 1.11 - 5.52 ) | 2.47 ( 5.26 ) | 2.47 ( 1.26 ) | 1.3 ( 0.21 ) |
| Lymphoedema | 5 | 29.81 ( 12.38 - 71.81 ) | 29.71 ( 138.42 ) | 29.64 ( 14.21 ) | 4.89 ( 3.71 ) |
| Metastases To Central Nervous System | 5 | 17.12 ( 7.11 - 41.22 ) | 17.06 ( 75.53 ) | 17.04 ( 8.17 ) | 4.09 ( 2.91 ) |
| Pneumothorax | 5 | 14.62 ( 6.07 - 35.19 ) | 14.57 ( 63.14 ) | 14.55 ( 6.98 ) | 3.86 ( 2.68 ) |
| Blood Sodium Decreased | 5 | 13.85 ( 5.75 - 33.36 ) | 13.81 ( 59.36 ) | 13.8 ( 6.61 ) | 3.79 ( 2.6 ) |
| Deafness | 5 | 9.02 ( 3.75 - 21.7 ) | 8.99 ( 35.48 ) | 8.98 ( 4.31 ) | 3.17 ( 1.99 ) |
| Taste Disorder | 5 | 5.68 ( 2.36 - 13.66 ) | 5.66 ( 19.19 ) | 5.66 ( 2.71 ) | 2.5 ( 1.32 ) |
| Renal Disorder | 5 | 5.1 ( 2.12 - 12.27 ) | 5.08 ( 16.4 ) | 5.08 ( 2.44 ) | 2.34 ( 1.16 ) |
| Respiratory Failure | 5 | 4.11 ( 1.71 - 9.89 ) | 4.1 ( 11.71 ) | 4.1 ( 1.96 ) | 2.03 ( 0.85 ) |
| Hepatic Enzyme Increased | 5 | 3.05 ( 1.27 - 7.35 ) | 3.05 ( 6.87 ) | 3.04 ( 1.46 ) | 1.61 ( 0.43 ) |
| Blood Albumin Decreased | 4 | 34.43 ( 12.89 - 92 ) | 34.34 ( 129.15 ) | 34.25 ( 15.05 ) | 5.1 ( 3.8 ) |
| Nephropathy Toxic | 4 | 15.64 ( 5.86 - 41.75 ) | 15.59 ( 54.58 ) | 15.58 ( 6.85 ) | 3.96 ( 2.67 ) |
| Hospice Care | 4 | 12.28 ( 4.6 - 32.79 ) | 12.25 ( 41.31 ) | 12.24 ( 5.38 ) | 3.61 ( 2.32 ) |
| Pericardial Effusion | 4 | 8.78 ( 3.29 - 23.44 ) | 8.76 ( 27.49 ) | 8.76 ( 3.85 ) | 3.13 ( 1.84 ) |
| Hepatotoxicity | 4 | 7.42 ( 2.78 - 19.79 ) | 7.4 ( 22.12 ) | 7.39 ( 3.25 ) | 2.89 ( 1.59 ) |
| Hepatic Function Abnormal | 4 | 4.84 ( 1.81 - 12.91 ) | 4.83 ( 12.14 ) | 4.82 ( 2.12 ) | 2.27 ( 0.98 ) |
| Swelling Face | 4 | 3.53 ( 1.32 - 9.43 ) | 3.53 ( 7.24 ) | 3.52 ( 1.55 ) | 1.82 ( 0.52 ) |
| Cardiac Failure | 4 | 2.49 ( 0.93 - 6.63 ) | 2.48 ( 3.54 ) | 2.48 ( 1.09 ) | 1.31 ( 0.02 ) |
| Malignant Pleural Effusion | 3 | 100.62 ( 32.27 - 313.72 ) | 100.41 ( 293.03 ) | 99.66 ( 38.49 ) | 6.64 ( 5.19 ) |
| Infectious Pleural Effusion | 3 | 74.29 ( 23.85 - 231.36 ) | 74.13 ( 215.24 ) | 73.72 ( 28.5 ) | 6.2 ( 4.75 ) |
| Organising Pneumonia | 3 | 24.32 ( 7.83 - 75.59 ) | 24.27 ( 66.82 ) | 24.23 ( 9.38 ) | 4.6 ( 3.15 ) |
| Musculoskeletal Chest Pain | 3 | 9.52 ( 3.07 - 29.58 ) | 9.51 ( 22.82 ) | 9.5 ( 3.68 ) | 3.25 ( 1.8 ) |
| Musculoskeletal Pain | 3 | 6.35 ( 2.05 - 19.73 ) | 6.34 ( 13.49 ) | 6.34 ( 2.46 ) | 2.66 ( 1.22 ) |
| Ascites | 3 | 5.44 ( 1.75 - 16.9 ) | 5.43 ( 10.85 ) | 5.43 ( 2.1 ) | 2.44 ( 1 ) |
| Urinary Retention | 3 | 4.73 ( 1.52 - 14.69 ) | 4.72 ( 8.81 ) | 4.72 ( 1.83 ) | 2.24 ( 0.79 ) |
| Cardiac Failure Congestive | 3 | 4.11 ( 1.32 - 12.76 ) | 4.1 ( 7.04 ) | 4.1 ( 1.59 ) | 2.04 ( 0.59 ) |
| Skin Discolouration | 3 | 3.42 ( 1.1 - 10.62 ) | 3.42 ( 5.13 ) | 3.42 ( 1.32 ) | 1.77 ( 0.33 ) |
| Liver Disorder | 3 | 3.23 ( 1.04 - 10.02 ) | 3.22 ( 4.6 ) | 3.22 ( 1.25 ) | 1.69 ( 0.24 ) |
| Rash Erythematous | 3 | 3.19 ( 1.03 - 9.9 ) | 3.18 ( 4.49 ) | 3.18 ( 1.23 ) | 1.67 ( 0.23 ) |
| Cellulitis | 3 | 3.15 ( 1.01 - 9.78 ) | 3.14 ( 4.39 ) | 3.14 ( 1.22 ) | 1.65 ( 0.21 ) |
| Device Related Thrombosis | 2 | 68.6 ( 17.08 - 275.55 ) | 68.5 ( 132.34 ) | 68.15 ( 21.29 ) | 6.09 ( 4.42 ) |
| Heart Valve Incompetence | 2 | 34.48 ( 8.6 - 138.24 ) | 34.43 ( 64.75 ) | 34.34 ( 10.74 ) | 5.1 ( 3.43 ) |
| Disease Complication | 2 | 21.01 ( 5.24 - 84.2 ) | 20.98 ( 38.01 ) | 20.95 ( 6.56 ) | 4.39 ( 2.72 ) |
| Hepatic Enzyme Abnormal | 2 | 18.71 ( 4.67 - 74.97 ) | 18.69 ( 33.43 ) | 18.66 ( 5.84 ) | 4.22 ( 2.55 ) |
| Renal Function Test Abnormal | 2 | 17.56 ( 4.38 - 70.34 ) | 17.53 ( 31.14 ) | 17.51 ( 5.48 ) | 4.13 ( 2.46 ) |
| Decubitus Ulcer | 2 | 14.77 ( 3.69 - 59.15 ) | 14.75 ( 25.6 ) | 14.73 ( 4.61 ) | 3.88 ( 2.21 ) |
| Erythema Multiforme | 2 | 12.93 ( 3.23 - 51.78 ) | 12.91 ( 21.96 ) | 12.9 ( 4.04 ) | 3.69 ( 2.02 ) |
| Blood Urea Increased | 2 | 10.25 ( 2.56 - 41.05 ) | 10.24 ( 16.66 ) | 10.23 ( 3.2 ) | 3.35 ( 1.69 ) |
| Electrolyte Imbalance | 2 | 8.57 ( 2.14 - 34.32 ) | 8.56 ( 13.35 ) | 8.55 ( 2.68 ) | 3.1 ( 1.43 ) |
| Pharyngitis Streptococcal | 2 | 7.87 ( 1.97 - 31.53 ) | 7.86 ( 11.98 ) | 7.86 ( 2.46 ) | 2.97 ( 1.31 ) |
| Ischaemic Stroke | 2 | 7.1 ( 1.77 - 28.42 ) | 7.09 ( 10.46 ) | 7.09 ( 2.22 ) | 2.82 ( 1.16 ) |
| Eye Infection | 2 | 6.92 ( 1.73 - 27.73 ) | 6.92 ( 10.12 ) | 6.91 ( 2.17 ) | 2.79 ( 1.12 ) |
| Gastric Ulcer | 2 | 6.6 ( 1.65 - 26.41 ) | 6.59 ( 9.48 ) | 6.59 ( 2.06 ) | 2.72 ( 1.05 ) |

Abbreviation: ROR, reporting odds ratio; PRR, proportional reporting ratio; EBGM, empirical Bayesian geometric mean; EBGM05, the lower limit of the 95% CI of EBGM; IC, information component; IC025, the lower limit of the 95% CI of the IC; CI, confidence interval; PT,preferred term.
