# Supplementary material for: Real-world safety of Tepotinib: Insights from the Food and Drug Administration Adverse Event Reporting System
Source: PLoS One. 2025 Dec 18;20(12):e0339005. doi: 10.1371/journal.pone.0339005 (PMC12714243; doi:10.1371/journal.pone.0339005)
Supplement: S4 Table — (DOCX) [file pone.0339005.s004.docx]

Supporting information

**S4 Table. Top 20 most common positive adverse events of Tepotinib in males at the PT level.**

| PT | Case numbers | ROR(95%CI) | PRR(χ^2^) | EBGM(EBGM05) | IC(IC025) |
| --- | --- | --- | --- | --- | --- |
| Death | 51 | 4.61 ( 3.46 - 6.14 ) | 4.31 ( 132.14 ) | 4.31 ( 3.39 ) | 2.11 ( 1.69 ) |
| Oedema Peripheral | 29 | 37.3 ( 25.68 - 54.19 ) | 35.61 ( 973.15 ) | 35.48 ( 25.96 ) | 5.15 ( 4.61 ) |
| Renal Impairment | 22 | 19.3 ( 12.61 - 29.54 ) | 18.65 ( 367.49 ) | 18.62 ( 13.04 ) | 4.22 ( 3.61 ) |
| Fatigue | 20 | 2.87 ( 1.84 - 4.48 ) | 2.81 ( 23.51 ) | 2.81 ( 1.93 ) | 1.49 ( 0.85 ) |
| Oedema | 18 | 47.19 ( 29.5 - 75.5 ) | 45.85 ( 786.57 ) | 45.64 ( 30.81 ) | 5.51 ( 4.84 ) |
| Disease Progression | 18 | 11.58 ( 7.25 - 18.52 ) | 11.28 ( 168.84 ) | 11.27 ( 7.61 ) | 3.49 ( 2.82 ) |
| Diarrhoea | 17 | 2.79 ( 1.72 - 4.51 ) | 2.74 ( 18.95 ) | 2.74 ( 1.83 ) | 1.45 ( 0.76 ) |
| Decreased Appetite | 15 | 6.36 ( 3.81 - 10.61 ) | 6.23 ( 66.01 ) | 6.22 ( 4.05 ) | 2.64 ( 1.91 ) |
| Peripheral Swelling | 13 | 8.24 ( 4.76 - 14.28 ) | 8.09 ( 80.91 ) | 8.08 ( 5.1 ) | 3.01 ( 2.23 ) |
| Nausea | 12 | 2.57 ( 1.45 - 4.55 ) | 2.54 ( 11.26 ) | 2.54 ( 1.57 ) | 1.34 ( 0.53 ) |
| Blood Creatinine Increased | 11 | 14.13 ( 7.78 - 25.65 ) | 13.89 ( 131.6 ) | 13.88 ( 8.42 ) | 3.79 ( 2.95 ) |
| Interstitial Lung Disease | 10 | 16.55 ( 8.85 - 30.93 ) | 16.3 ( 143.49 ) | 16.27 ( 9.64 ) | 4.02 ( 3.15 ) |
| Pruritus | 10 | 2.8 ( 1.5 - 5.23 ) | 2.77 ( 11.37 ) | 2.77 ( 1.64 ) | 1.47 ( 0.59 ) |
| Constipation | 8 | 3.94 ( 1.96 - 7.92 ) | 3.9 ( 17.34 ) | 3.9 ( 2.18 ) | 1.96 ( 1 ) |
| Fluid Retention | 7 | 16.64 ( 7.9 - 35.08 ) | 16.47 ( 101.6 ) | 16.44 ( 8.81 ) | 4.04 ( 3.01 ) |
| Hypoalbuminaemia | 6 | 66.05 ( 29.48 - 147.98 ) | 65.42 ( 378.13 ) | 64.99 ( 33.09 ) | 6.02 ( 4.92 ) |
| Pleural Effusion | 6 | 10.05 ( 4.5 - 22.47 ) | 9.96 ( 48.38 ) | 9.95 ( 5.08 ) | 3.32 ( 2.22 ) |
| Pulmonary Toxicity | 5 | 44.67 ( 18.49 - 107.92 ) | 44.32 ( 210.8 ) | 44.13 ( 21.09 ) | 5.46 ( 4.28 ) |
| Renal Disorder | 4 | 7.76 ( 2.9 - 20.74 ) | 7.71 ( 23.37 ) | 7.71 ( 3.38 ) | 2.95 ( 1.65 ) |
| Feeling Abnormal | 4 | 2.52 ( 0.94 - 6.73 ) | 2.51 ( 3.64 ) | 2.51 ( 1.1 ) | 1.33 ( 0.03 ) |

Abbreviation: ROR, reporting odds ratio; PRR, proportional reporting ratio; EBGM, empirical Bayesian geometric mean; EBGM05, the lower limit of the 95% CI of EBGM; IC, information component; IC025, the lower limit of the 95% CI of the IC; CI, confidence interval; PT,preferred term; AEs, adverse event.
