# Supplementary material for: Real-world safety of Tepotinib: Insights from the Food and Drug Administration Adverse Event Reporting System
Source: PLoS One. 2025 Dec 18;20(12):e0339005. doi: 10.1371/journal.pone.0339005 (PMC12714243; doi:10.1371/journal.pone.0339005)
Supplement: S5 Table — (DOCX) [file pone.0339005.s005.docx]

Supporting information

**S5 Table. Top 20 most common positive adverse events of Tepotinib in females at the PT level.**

| PT | Case numbers | ROR(95%CI) | PRR(χ^2^) | EBGM(EBGM05) | IC(IC025) |
| --- | --- | --- | --- | --- | --- |
| Death | 47 | 6.92 ( 5.15 - 9.3 ) | 6.54 ( 222.61 ) | 6.54 ( 5.1 ) | 2.71 ( 2.28 ) |
| Diarrhoea | 33 | 4.06 ( 2.86 - 5.76 ) | 3.92 ( 72.66 ) | 3.92 ( 2.93 ) | 1.97 ( 1.47 ) |
| Oedema Peripheral | 31 | 37.13 ( 25.9 - 53.23 ) | 35.61 ( 1041.23 ) | 35.52 ( 26.28 ) | 5.15 ( 4.63 ) |
| Nausea | 25 | 2.59 ( 1.74 - 3.87 ) | 2.54 ( 23.65 ) | 2.54 ( 1.82 ) | 1.34 ( 0.77 ) |
| Fatigue | 24 | 2.36 ( 1.57 - 3.54 ) | 2.32 ( 18.18 ) | 2.31 ( 1.65 ) | 1.21 ( 0.62 ) |
| Oedema | 22 | 39.62 ( 25.9 - 60.59 ) | 38.46 ( 800.99 ) | 38.35 ( 26.88 ) | 5.26 ( 4.65 ) |
| Disease Progression | 21 | 16.6 ( 10.75 - 25.62 ) | 16.15 ( 298.66 ) | 16.13 ( 11.22 ) | 4.01 ( 3.39 ) |
| Renal Impairment | 19 | 24.04 ( 15.24 - 37.93 ) | 23.44 ( 408 ) | 23.41 ( 15.98 ) | 4.55 ( 3.89 ) |
| Blood Creatinine Increased | 12 | 23.86 ( 13.48 - 42.23 ) | 23.48 ( 258.04 ) | 23.44 ( 14.54 ) | 4.55 ( 3.74 ) |
| Peripheral Swelling | 12 | 4.64 ( 2.62 - 8.21 ) | 4.58 ( 33.69 ) | 4.58 ( 2.84 ) | 2.19 ( 1.39 ) |
| Vomiting | 11 | 2.01 ( 1.11 - 3.64 ) | 1.99 ( 5.47 ) | 1.99 ( 1.21 ) | 0.99 ( 0.15 ) |
| Swelling | 9 | 5.99 ( 3.1 - 11.57 ) | 5.93 ( 36.96 ) | 5.93 ( 3.42 ) | 2.57 ( 1.65 ) |
| Weight Increased | 8 | 3.1 ( 1.55 - 6.23 ) | 3.08 ( 11.29 ) | 3.08 ( 1.72 ) | 1.62 ( 0.66 ) |
| Decreased Appetite | 8 | 2.91 ( 1.45 - 5.84 ) | 2.89 ( 9.92 ) | 2.89 ( 1.61 ) | 1.53 ( 0.56 ) |
| Alopecia | 7 | 2.37 ( 1.13 - 5 ) | 2.36 ( 5.51 ) | 2.36 ( 1.27 ) | 1.24 ( 0.21 ) |
| Generalised Oedema | 7 | 60.8 ( 28.83 - 128.2 ) | 60.23 ( 405.95 ) | 59.96 ( 32.12 ) | 5.91 ( 4.88 ) |
| Abdominal Pain Upper | 6 | 2.37 ( 1.06 - 5.29 ) | 2.36 ( 4.7 ) | 2.36 ( 1.2 ) | 1.24 ( 0.14 ) |
| Pleural Effusion | 6 | 11.56 ( 5.17 - 25.82 ) | 11.47 ( 57.34 ) | 11.46 ( 5.85 ) | 3.52 ( 2.42 ) |
| Deafness | 5 | 17.62 ( 7.31 - 42.47 ) | 17.5 ( 77.73 ) | 17.48 ( 8.37 ) | 4.13 ( 2.94 ) |
| Pulmonary Oedema | 5 | 12.6 ( 5.23 - 30.37 ) | 12.52 ( 52.97 ) | 12.51 ( 5.99 ) | 3.64 ( 2.46 ) |

Abbreviation: ROR, reporting odds ratio; PRR, proportional reporting ratio; EBGM, empirical Bayesian geometric mean; EBGM05, the lower limit of the 95% CI of EBGM; IC, information component; IC025, the lower limit of the 95% CI of the IC; CI, confidence interval; PT,preferred term; AEs, adverse events.
