# Supplementary material for: Real-world safety of Tepotinib: Insights from the Food and Drug Administration Adverse Event Reporting System
Source: PLoS One. 2025 Dec 18;20(12):e0339005. doi: 10.1371/journal.pone.0339005 (PMC12714243; doi:10.1371/journal.pone.0339005)
Supplement: S6 Table — (DOCX) [file pone.0339005.s006.docx]

Supporting information

**S6 Table. Top 20 most common positive adverse events of Tepotinib in patients under 75 years at the PT level.**

| **PT** | **Case numbers** | **ROR(95%CI)** | **PRR(χ^2^)** | **EBGM(EBGM05)** | **IC(IC025)** |
| --- | --- | --- | --- | --- | --- |
| Death | 23 | 6.43 ( 4.23 - 9.79 ) | 6.15 ( 99.96 ) | 6.15 ( 4.33 ) | 2.62 ( 2.01 ) |
| Disease Progression | 18 | 18.49 ( 11.54 - 29.64 ) | 17.77 ( 285.38 ) | 17.76 ( 11.97 ) | 4.15 ( 3.47 ) |
| Fatigue | 16 | 2.97 ( 1.8 - 4.89 ) | 2.9 ( 20.15 ) | 2.9 ( 1.91 ) | 1.54 ( 0.82 ) |
| Diarrhoea | 15 | 3.28 ( 1.96 - 5.49 ) | 3.2 ( 22.94 ) | 3.2 ( 2.08 ) | 1.68 ( 0.94 ) |
| Nausea | 15 | 2.91 ( 1.74 - 4.88 ) | 2.85 ( 18.21 ) | 2.85 ( 1.85 ) | 1.51 ( 0.78 ) |
| Oedema | 12 | 36.41 ( 20.5 - 64.64 ) | 35.44 ( 401.24 ) | 35.38 ( 21.88 ) | 5.14 ( 4.33 ) |
| Oedema Peripheral | 11 | 21.48 ( 11.8 - 39.1 ) | 20.97 ( 209.23 ) | 20.95 ( 12.69 ) | 4.39 ( 3.54 ) |
| Renal Impairment | 11 | 19.54 ( 10.74 - 35.56 ) | 19.08 ( 188.5 ) | 19.06 ( 11.55 ) | 4.25 ( 3.41 ) |
| Peripheral Swelling | 9 | 7.09 ( 3.66 - 13.72 ) | 6.96 ( 46.09 ) | 6.96 ( 4.01 ) | 2.8 ( 1.88 ) |
| Vomiting | 8 | 2.51 ( 1.25 - 5.05 ) | 2.48 ( 7.13 ) | 2.48 ( 1.38 ) | 1.31 ( 0.34 ) |
| Blood Creatinine Increased | 7 | 16.14 ( 7.65 - 34.06 ) | 15.9 ( 97.74 ) | 15.88 ( 8.5 ) | 3.99 ( 2.96 ) |
| Decreased Appetite | 7 | 4.43 ( 2.10 - 9.34 ) | 4.37 ( 18.26 ) | 4.37 ( 2.34 ) | 2.13 ( 1.1 ) |
| Pruritus | 7 | 2.27 ( 1.07 - 4.78 ) | 2.25 ( 4.87 ) | 2.25 ( 1.20 ) | 1.17 ( 0.14 ) |
| Interstitial Lung Disease | 5 | 15.53 ( 6.43 - 37.5 ) | 15.36 ( 67.13 ) | 15.35 ( 7.34 ) | 3.94 ( 2.75 ) |
| Swelling | 5 | 6.91 ( 2.86 - 16.68 ) | 6.84 ( 24.96 ) | 6.84 ( 3.27 ) | 2.77 ( 1.59 ) |
| Alopecia | 4 | 3.32 ( 1.24 - 8.88 ) | 3.30 ( 6.42 ) | 3.3 ( 1.45 ) | 1.72 ( 0.42 ) |
| Alanine Aminotransferase Increased | 4 | 10.1 ( 3.77 - 27.03 ) | 10.01 ( 32.47 ) | 10.01 ( 4.39 ) | 3.32 ( 2.03 ) |
| Constipation | 4 | 2.93 ( 1.1 - 7.85 ) | 2.92 ( 5.05 ) | 2.92 ( 1.28 ) | 1.54 ( 0.25 ) |
| Hypoalbuminaemia | 4 | 63.38 ( 23.65 - 169.87 ) | 62.81 ( 242.63 ) | 62.63 ( 27.45 ) | 5.97 ( 4.67 ) |
| Anaemia | 4 | 3.51 ( 1.31 - 9.41 ) | 3.49 ( 7.13 ) | 3.49 ( 1.53 ) | 1.80 ( 0.51 ) |

Abbreviation: ROR, reporting odds ratio; PRR, proportional reporting ratio; EBGM, empirical Bayesian geometric mean; EBGM05, the lower limit of the 95% CI of EBGM; IC, information component; IC025, the lower limit of the 95% CI of the IC; CI, confidence interval; PT,preferred term; AEs, adverse events.
