# Supplementary material for: Real-world safety of Tepotinib: Insights from the Food and Drug Administration Adverse Event Reporting System
Source: PLoS One. 2025 Dec 18;20(12):e0339005. doi: 10.1371/journal.pone.0339005 (PMC12714243; doi:10.1371/journal.pone.0339005)
Supplement: S7 Table — (DOCX) [file pone.0339005.s007.docx]

Supporting information

**S7 Table. Top 20 most common positive adverse events of Tepotinib in patients aged 75 years and over at the PT level.**

| PT | Case numbers | ROR(95%CI) | PRR(χ^2^) | EBGM(EBGM05) | IC(IC025) |
| --- | --- | --- | --- | --- | --- |
| Death | 55 | 2.45 ( 1.86 - 3.23 ) | 2.34 ( 43.68 ) | 2.34 ( 1.86 ) | 1.23 ( 0.83 ) |
| Oedema Peripheral | 35 | 19.43 ( 13.82 - 27.33 ) | 18.55 ( 578.06 ) | 18.41 ( 13.84 ) | 4.2 ( 3.71 ) |
| Diarrhoea | 32 | 3.11 ( 2.18 - 4.44 ) | 3.02 ( 43.82 ) | 3.02 ( 2.24 ) | 1.59 ( 1.08 ) |
| Fatigue | 26 | 2.6 ( 1.76 - 3.85 ) | 2.54 ( 24.7 ) | 2.54 ( 1.83 ) | 1.35 ( 0.78 ) |
| Renal Impairment | 25 | 13.44 ( 9.01 - 20.05 ) | 13.02 ( 276.48 ) | 12.95 ( 9.27 ) | 3.69 ( 3.12 ) |
| Oedema | 22 | 33.43 ( 21.81 - 51.25 ) | 32.46 ( 662.04 ) | 32.02 ( 22.4 ) | 5 ( 4.39 ) |
| Nausea | 22 | 3.1 ( 2.03 - 4.75 ) | 3.04 ( 30.39 ) | 3.04 ( 2.13 ) | 1.6 ( 0.99 ) |
| Decreased Appetite | 15 | 3.43 ( 2.05 - 5.72 ) | 3.38 ( 25.21 ) | 3.37 ( 2.2 ) | 1.75 ( 1.02 ) |
| Peripheral Swelling | 14 | 4.63 ( 2.73 - 7.86 ) | 4.56 ( 38.98 ) | 4.55 ( 2.92 ) | 2.19 ( 1.43 ) |
| Blood Creatinine Increased | 11 | 10.49 ( 5.77 - 19.05 ) | 10.35 ( 92.59 ) | 10.3 ( 6.25 ) | 3.37 ( 2.52 ) |
| Pleural Effusion | 10 | 9.14 ( 4.89 - 17.08 ) | 9.03 ( 71.21 ) | 9 ( 5.33 ) | 3.17 ( 2.29 ) |
| Disease Progression | 9 | 6.09 ( 3.15 - 11.76 ) | 6.03 ( 37.71 ) | 6.01 ( 3.47 ) | 2.59 ( 1.67 ) |
| Constipation | 9 | 2.24 ( 1.16 - 4.33 ) | 2.23 ( 6.12 ) | 2.23 ( 1.28 ) | 1.15 ( 0.24 ) |
| Weight Increased | 8 | 5.97 ( 2.97 - 12 ) | 5.92 ( 32.66 ) | 5.9 ( 3.29 ) | 2.56 ( 1.59 ) |
| Fluid Retention | 7 | 10.13 ( 4.81 - 21.36 ) | 10.04 ( 56.82 ) | 10.01 ( 5.36 ) | 3.32 ( 2.29 ) |
| Interstitial Lung Disease | 7 | 5.9 ( 2.8 - 12.44 ) | 5.86 ( 28.16 ) | 5.84 ( 3.13 ) | 2.55 ( 1.52 ) |
| Dry Skin | 6 | 6.66 ( 2.98 - 14.9 ) | 6.62 ( 28.56 ) | 6.6 ( 3.37 ) | 2.72 ( 1.62 ) |
| Abdominal Pain Upper | 5 | 2.42 ( 1 - 5.82 ) | 2.41 ( 4.12 ) | 2.4 ( 1.15 ) | 1.27 ( 0.08 ) |
| Swelling | 5 | 5.85 ( 2.42 - 14.11 ) | 5.82 ( 19.91 ) | 5.8 ( 2.78 ) | 2.54 ( 1.35 ) |
| Taste Disorder | 5 | 8.19 ( 3.39 - 19.78 ) | 8.14 ( 31.25 ) | 8.12 ( 3.88 ) | 3.02 ( 1.84 ) |

Abbreviation: ROR, reporting odds ratio; PRR, proportional reporting ratio; EBGM, empirical Bayesian geometric mean; EBGM05, the lower limit of the 95% CI of EBGM; IC, information component; IC025, the lower limit of the 95% CI of the IC; CI, confidence interval; PT,preferred term; AEs, adverse event.
